# Evolution of morphological but not aggressiveness‐related traits following a major resistance breakdown in the poplar rust fungus, *Melampsora larici‐populina*

**DOI:** 10.1111/eva.13136

**Published:** 2020-10-16

**Authors:** Agathe Maupetit, Bénédicte Fabre, Jérémy Pétrowski, Axelle Andrieux, Stéphane De Mita, Pascal Frey, Fabien Halkett, Katherine J. Hayden

**Affiliations:** ^1^ INRAE Université de Lorraine Nancy France; ^2^ Royal Botanical Garden Edinburgh Edinburgh UK; ^3^ Present address: IFREMER, Physiology and Biotechnology of Algae Laboratory Nantes France

**Keywords:** disease‐associated traits, heritability, mixed model, plant pathogen, *Q*_ST_‐*F*_ST_ comparisons, temporal sampling

## Abstract

Crop varieties carrying qualitative resistance to targeted pathogens lead to strong selection pressure on parasites, often resulting in resistance breakdown. It is well known that qualitative resistance breakdowns modify pathogen population structure but few studies have analyzed the consequences on their quantitative aggressiveness‐related traits. The aim of this study was to characterize the evolution of these traits following a resistance breakdown in the poplar rust fungus, *Melampsora larici‐populina*. We based our experiment on three temporal populations sampled just before the breakdown event, immediately after and four years later. First, we quantified phenotypic differences among populations for a set of aggressiveness traits on a universally susceptible cultivar (infection efficiency, latent period, lesion size, mycelium quantity, and sporulation rate) and one morphological trait (mean spore volume). Then, we estimated heritability to establish which traits could be subjected to adaptive evolution and tested for evidence of selection. Our results revealed significant changes in the morphological trait but no variation in aggressiveness traits. By contrast, recent works have demonstrated that quantitative resistance (initially assumed more durable) could be eroded and lead to increased aggressiveness. Hence, this study is one example suggesting that the use of qualitative resistance may be revealed to be less detrimental to long‐term sustainable crop production.

## 1 INTRODUCTION

Plant pathogens are one of the main constraints on production in agricultural ecosystems. Yet heavy pesticide use can cause environmental harm. An alternative solution to control pathogens is to breed resistant crop varieties, reconciling agricultural production, and environmental health. Breeders thus select crop cultivars carrying qualitative or quantitative resistance factors to several targeted pathogens. However, human activities including resistance breeding are known to drive the evolution of plant pathogens in agro‐systems by shaping genetically homogeneous host populations and creating environments conducive to pathogen spread (Papaïx et al., 2018; Pariaud et al., 2009; Stukenbrock & McDonald, 2008; Zhan et al., 2015).

Qualitative resistance represents the main and best characterized selective pressure exerted by crop species on pathogen populations. Cultivars carrying qualitative resistance genes prevent any infection by specific strains of the pathogen. This type of resistance often relies on the gene‐for‐gene model (Flor, 1971) and thus usually has a simple genetic determinism with a single gene conferring resistance to the host. Virulence or avirulence of pathogens is determined by the absence or presence, respectively, of an effector protein encoded by an avirulence allele, which is the counterpart to the resistance allele. Combined with a homogenous host population at the scale of an agro‐system, this simple genetic system favors rapid resistance breakdowns through the emergence of new virulence factors (Stukenbrock & McDonald, 2008). Resistance breakdowns and their consequences on genetic diversity are well documented (Brown & Tellier, 2011; Zhan et al., 2015) but little is known about the consequences on quantitative traits of the pathogen, such as aggressiveness, which are those quantitative traits that can be measured from disease symptoms (Pariaud et al., 2009) and morphological traits.

One important repercussion of a qualitative resistance breakdown is the colonization of new pools of hosts, which were previously resistant to the pathogen. This process corresponds to a host range expansion. It involves two main steps: first, the early infection of the new host with a necessary adaptation to physiological characteristics differing from the original host; second, the subsequent invasion of an ecological niche, implying pathogen population dispersal and increased competition among individuals. In these respects, Lê Van et al. (2012) suggested that host range expansion can lead to specialization or speciation. Indeed, the gain of new virulence is known to impact the pathogen's demography, sometimes leading to the emergence of new crop pathogen species (Couch et al., 2005; Stukenbrock et al., 2007). In the course of this new interaction, any number of quantitative traits may be differently expressed, including those related to aggressiveness.

Taken together, these disruptions are expected to trigger modifications at a phenotypic level, potentially influenced by the existence of trade‐offs. In order to understand the evolution of parasites facing qualitative resistance, we investigated the consequences of a resistance breakdown at a phenotypic level on both aggressiveness and morphological traits.

In this study, we focus on the poplar rust fungus, *Melampsora larici‐populina*, a foliar pathogen which has overcome eight qualitative resistance types since 1949 (Pinon & Frey, 2005) including one well studied resistance breakdown event in 1994. Because *M. larici‐populina* is the most important threat to poplar cultivation in Europe, poplar cultivars carrying qualitative resistance have been released in commercial cultivation for more than 70 years. In 1982, the poplar cultivar *Populus* × *interamericana* ‘Beaupré’ which carries the resistance denoted as R7 was released throughout Europe. This cultivar remained immune to *M. larici‐populina* for 12 years but, in 1994, this resistance was overcome. The first corresponding virulent “7” strains of *M. larici‐populina* were observed in September 1994 in Belgium, rapidly followed by its observation in northern France (Pinon & Frey, 2005). These virulent “7” strains most likely originated from the local avirulent “7” population (Persoons et al., 2017). The new virulence triggered the colonization of the ecological niche formed by ‘Beaupré’ stands. In less than five years, virulent “7” strains spread all over France, reaching the southern regions. This population rapidly superseded the initial avirulent “7” population where it was present before the resistance R7 breakdown, even in poplar stands not carrying R7.

The objective of this study was to characterize the evolution of aggressiveness and morphological traits following the resistance R7 breakdown event. To tackle this issue, we consider three populations of *M. larici‐populina* from a historical collection, sampled before, just after the resistance R7 breakdown and four years after. This temporal sampling allows us to answer the following questions: (a) Is there differentiation between traits before and after the resistance breakdown? (b) Which traits could respond to selection? (c) What type of selection was applied on these traits? And (d) are there genetic trade‐offs shaping the joint evolution of multiple traits? To answer question (a), we assessed—using linear regressions—the differentiation of aggressiveness traits among the three populations. To address question (b), we estimated the genetic determinism of each trait through the computation of heritability. For question (c), to find out the type of selection applied on the traits, we compared the variances within and between populations of neutral markers and quantitative traits, that is, we performed *Q*_ST_–*F*_ST_ comparisons. Finally, to answer question (d), we searched for genetic trade‐offs through the analysis of genetic covariances between traits within each population.

## 2 MATERIALS AND METHODS

### 2.1 Plant and fungal material

We studied 54 strains from a historical collection of *M. larici‐populina* that has been cryopreserved at −80°C at INRAE in Nancy (France) giving access to fixed genotypes. All strains were sampled in northern France and in Belgium among homogenous pathogen populations. Three temporal populations of 18 strains each were chosen to reflect three key dates (see Table S1 for population description): population 1993 was collected on susceptible poplars before the resistance R7 breakdown; populations 1994 and 1998 were collected on resistant R7 poplars, just after the resistance breakdown and four years later, respectively. Population 1993 originated from a single location in Northeastern France (Amance). Population 1994 comprises French and Belgian strains (the Belgian strains originated from the location where the first virulent “7” isolates were detected). Population 1998 was sampled in two locations, in Amance and in Moÿ‐de‐l’Aisne (Northern France, some 240 km apart).

All *M. larici‐populina* strains were inoculated on the same cultivar to carry out a common garden experiment. Characterization of quantitative traits was thus performed on excised leaf disks of *Populus deltoides × P. nigra* ‘Robusta’ which is known to be susceptible to all tested strains (Pinon & Frey, 2005). Poplar plants were grown from dormant cuttings in 10‐L pots containing a sand‐peat (1:1, v/v) mixture, with an initial fertilization of 3.5 g/L CaCO_3_ and 6 g/L of slow release 13–13–13 N‐P‐K fertilizer (Nutricote® T100, Fertil). The plants were grown in a growth chamber (5.7 plants/m^2^) regulated at 20/22°C (night/day temperatures) and 16 hr photoperiod, and were watered daily with deionized water. After four months, young trees were about 1.2 m high and exhibited 25 to 30 fully expanded leaves.

As leaf age and physiology are known to influence trait variation (Maupetit et al., 2018; Sharma et al., 1980), we used leaves of the same leaf plastochron index (LPI, Larson & Isebrands, 1971). The common LPI was chosen to be after the transition from sink to source, which in poplar is established around LPI 6 (Coleman, 1986), and before any leaf senescence. Leaves of LPI 9 from 39 plants were thus used and harvested the day of inoculation. Forty‐eight 12‐mm‐diameter disks were excised from each poplar leaf and placed in flotation on deionized water in two 24‐well polystyrene cell culture plates.

A germination test was performed just before inoculation to ensure the quality of spores used (Pei et al., 2002). To this aim, spores of all strains were dispersed on the surface of a Petri dish containing agar (20 g/L). After an overnight incubation at 19 ± 1°C, the proportion of germinated spores was evaluated under a light microscope (100× magnification). All of the 54 strains had a germination rate higher than 90%, ensuring that the spores used for single‐spore inoculations were highly viable. Differences in infection efficiency can thus be attributed only to variations in aggressiveness (failure to infect, not to germinate). All *M. larici‐populina* strains were multiplied on poplar leaf disks prior to their inoculation.

### 2.2 Inoculation protocol and experimental design

We used a single‐spore inoculation protocol: A unique spore is picked with an eyelash and deposited in the center of an excised poplar leaf disk as described in Maupetit et al. (2018). This protocol has several advantages compared with traditional inoculation of foliar pathogens, which usually consists of spraying or depositing a suspension of spores. First, inoculum density is controlled so that lesion density and infection efficiency cannot influence trait values (Lannou, 2012; Pariaud et al., 2009). Second, the single‐spore inoculation protocol allows each lesion to be monitored separately—instead of a population of lesions as in traditional inoculation—giving access to the individual contribution to overall trait variation. This allows the estimation of heritability. This protocol is tedious but has been revealed to be assess reaction norms and trait variations along a gradient of leaf maturity (Maupetit et al., 2018).

The experimental design is crucial in common garden experiments (Kawecki & Ebert, 2004). All isolates must experience the same environment to remove putative confounding factors. Here, we used the inoculation plate (containing 24 leaf disks) as the statistical block. Strain replicates were arranged among inoculation plates to minimize confounding experimental effects. Each plate contained six replicates of four strains. Each strain was inoculated on at least 72 leaf disks (corresponding to 12 quarter‐plates). The replicates were distributed according to a balanced incomplete block (BIB) design in order to optimize the distribution of strain replicates among inoculation plates. Because the single‐spore inoculation protocol is highly time‐consuming, we were not able to inoculate more than nine plates per inoculation day per manipulator. Two manipulators inoculated the plates. Inoculations were thus distributed over fourteen days (each inoculation day formed a series of eighteen culture plates). Inoculation plates determined by the BIB design were distributed to manipulators and across inoculation dates to ensure equal repartition of strain replicates among experimental factors. The overall experiment consisted in 3,888 inoculated leaf disks.

Measurement of quantitative traits was conducted on 4 to 42 replicates per strain (mean number of replicates = 20), depending on strain infection success (mean infection success = 26%). One of the 54 strains failed to infect all 72 leaf disks and was thus removed from the analysis.

### 2.3 Quantitative trait measurements

All aggressiveness traits classically studied in plant–pathogen systems were measured: infection efficiency, latent period, lesion size, and sporulation rate (Lannou, 2012). Infection efficiency is defined as the probability that a spore creates a lesion. Latent period is the time between spore inoculation and the sporulation from this infection. Lesion size (also referred to as uredinia size for rust fungi) is defined as the surface area that produces spores (Kolmer & Leonard, 1986; Robert et al., 2004). Sporulation rate is defined as the number of spores produced by the lesion per unit of time (Clifford & Clothier, 1974; Kardin & Groth, 1989; Leonard, 1969). Beyond these four classical traits, we also measured the quantity of mycelium *in planta* developing in an infected leaf disk, through qPCR analysis. We measured one morphological trait: The mean volume of each spore, which was computed from length and width of spores, obtained from image analysis. All details of quantitative traits measurement are described in Maupetit et al. (2018).

In short, we monitored the infections for 13 days after spore inoculation, allowing measurements of latent period and infection efficiency. Emergence of uredinia was scored twice a day, at 9 a.m. and at 4 p.m. Infection efficiency was assessed for each leaf disk and was recorded as a binary variable: 1 if the deposited urediniospore produced one uredinium, 0 if not. At day 13 (end of the monitoring period), leaf disks were harvested to prevent the emergence of secondary lesions. The first step of the harvest consisted of separating the urediniospores produced from the leaf disk. Infected leaf disks were placed in 2 ml Eppendorf tubes with 2 ml of Isoton^®^ II isotonic buffered diluent (Beckman Coulter). The tubes were vigorously shaken for 20 s at 4 m/s on a MP FastPrep^®^‐24 homogenizer, in order to release all urediniospores from the uredinia. The second step consisted of measuring the size of the uredinia: photographs of the spore‐free uredinia were taken under a stereomicroscope (25 x magnification) coupled to a digital camera. To measure the number of yellow pixels corresponding to the sporulating area, pictures were analyzed using ImageJ version 1.5i with a dedicated script (Maupetit et al., 2018). Last, infected leaf disks were stored at −80°C and lyophilized before DNA extraction.

Measurements of sporulation rate and spore morphological parameters were performed on the spore suspensions using an Occhio® Flowcell FC200+ optical morphogranulometer, which allows simultaneous particle counting and image analysis of the particles. Before counting, spore suspensions were shaken to disperse clusters of spores. For each sample, counting was performed using the following settings: 0.15 ml of priming, 0.85 ml of volume analysis, and 7% of volume sampling (i.e., 7% within 0.85 ml were really analyzed, evenly distributed along the analyzed volume). To distinguish spores from other particles, a custom filter was applied on parameter values:

- Feret diameter length between 23 and 200 µm;
- bluntness between 0.6 and 1;
- minimum Feret diameter between 13.5 and 70 µm;
- eccentricity higher than 0.1;
- ellipse elongation between 0.18 and 0.79;
- width of ellipse minor axis lower than 22 µm;
- length of ellipse minor axis lower than 54 µm.

This filter was applied to the raw data to compute sporulation rate (expressed in spores lesion^−1^ day^−1^) and the dimensions (length and width) of spores produced by each uredinium. The volume of each spore was computed from the formula of the volume of an ellipsoid (Philibert et al., 2011):

$$\text{Spore volume}=\frac{4}{3}\pi \times \frac{\text{length}}{2}\times {\left(\frac{\text{width}}{2}\right)}^{2}$$

Spore volume is reported as the per‐sample mean.

### 2.4 DNA extraction and qPCR quantification of *in planta* mycelium

DNA was extracted from infected poplar leaf disks in 96‐well plates using the DNeasy96® DNA plant kit (Qiagen) as described in Maupetit et al. (2018). The infected leaf disks were randomly arranged in 38 half plates, keeping together all replicates from the same culture plate, to maintain statistical blocks. All DNA extraction plates were stored at −20°C until qPCR analysis. In order to correct for qPCR variance, three technical replicates of each DNA extraction plate were performed using an incomplete block design over 15 qPCR runs, each including a different combination of four DNA extraction plates (Figure S1).

### 2.5 Data analysis

Uneven infection efficiency led to large differences in the representation of strains within and among inoculation plates. In order to balance the experimental design and the number of replicates for all strains, 1,000 bootstrapped datasets were created by randomly selecting one replicate per strain and per plate. These bootstrapped datasets were used to compute phenotypic differentiation between populations, *Q*_ST_, broad‐sense heritability (*H* ^2^) and their confidence intervals.

To analyze the phenotypic differentiation of measured traits among populations, we used nonlinear mixed models with the following formula:

$$Y_{\mathrm{ijkl}}=\mu +{\text{Pop}}_{i}+{\text{Strain}}_{j}\left({\text{Pop}}_{i}\right)+\left({\text{day}}_{k}\times {\text{plate}}_{l}\right)+\varepsilon_{\mathrm{ijkl}}$$

where *Y_ijkl_* is the phenotype of the replicate *l* transformed to better fit a normal distribution (see Table 1 for transformation types). All variables were considered as random effects to estimate variance components (see below). Pop*_i_* is the population level (*i* = 1…3), with ${\text{Pop}}_{i}∼N\left(0,\sigma_{\mathrm{Gb}}^{2}\right)$. Strains_j_ (*j* = 1…18) are nested within populations, with ${\text{Strain}}_{j}∼N\left(0,\sigma_{\mathrm{Gw}}^{2}\right)$. Last, we considered the interaction between the two experimental effects, the day of inoculation, ${\text{day}}_{k}∼N\left(0,\sigma_{\text{day}}^{2}\right)$ with (*k* = 1…14) and inoculation plates ${\text{plate}}_{l}∼N\left(0,\sigma_{\text{plate}}^{2}\right)$ with (*l* = 1…240). Population differentiation was assessed with an analysis of variance contrasting the previous model and the null model (without population effect), with Wald chi‐square tests and α = 0.05. Differences of trait means were assessed with Tukey's HSD (honestly significant difference) test. Variance estimates were extracted from the null model for heritability computation and from the full model for *Q*_ST_ computation.

**Table 1:** Variable transformations and error families used

Aggressiveness trait	Variable transformation	Error family
Infection efficiency	None (identity)	Binomial
Latent period	None (identity)	Gamma
Lesion size	Square root	Gaussian
Mycelium quantity	Square root	Gaussian
Spore number	None (identity)	Gaussian
Sporulation rate	Cube root	Gaussian
Spore volume	Cube root	Gaussian

Negative genetic correlations between traits were considered as genetic trade‐offs. These were estimated from adjusted trait means for experimental effects for each strain: “day of inoculation,” “plant,” and “plate” nested in “day of inoculation” and “manipulator” (see Table 1 for transformations and error family). To ease understanding of the sign of genetic correlations, development speed (defined as the inverse of latent period) was considered instead of latent period.

Genetic correlations were tested with the Spearman correlation coefficient between each pair of traits. Significance was judged using Student's test, with *P*‐values adjusted using a false discovery rate correction for multiple tests with a threshold of 5%.

All analyses were conducted using R version 3.5.0, with packages lme4 (Bates, Mächler, Bolker, & Walker, 2015), car (Fox & Weisberg, 2019), sciplot (Morales, 2017), agricolae (Mendiburu, 2019), Hmisc (Harrell Jr., & Dupont, 2019), ade4 (Chessel, Dufour, & Thioulouse, 2004, p. 4), lsmeans (Lenth, 2018), lmerTest (Kuznetsova et al., 2017), and corrplot (Wei & Simko, 2017).

### 2.6 Heritability and population differentiation indices

The broad‐sense heritability (*H* ^2^) of each quantitative trait was computed for each population separately as defined by Nyquist and Baker (1991):

$$H^{2}=\frac{\sigma_{\text{Gw}}^{2}}{\sigma_{\text{Gw}}^{2}+\frac{\sigma_{\text{plate}}^{2}}{\text{plate}}+\frac{\sigma_{\text{day}}^{2}}{\text{day}}+\frac{\sigma_{\text{residuals}}^{2}}{\text{plate}\times \text{repetition}}}$$

where *σ* ^2^_Gw_, *σ* ^2^_plate_, *σ* ^2^_day_, and *σ* ^2^_residuals_ are the variance effects extracted from the null model (see section 2.5 Data analysis); plate is the mean number of plates with sporulating lesions from one strain; day is the mean number of days with positive infecting spores from one strain; repetition is the mean number of sporulating lesions from one strain from one plate (equal to 1 per definition in all bootstrapped datasets). Variances were estimated from the null model described above. Differences in heritability among traits and population were tested with a Kruskal–Wallis test followed by a post‐hoc Dunn test.

The fixation index, *F*_ST_, was computed on neutral markers assayed for each strain of the three populations using fstat version 2.9.3 (Goudet, 1995). Strains were genotyped using a set of 25 microsatellite markers. Genotype data were available from a previous population genetic study (data archive available from Dryad repository: https://doi.org/10.5061/dryad.r6d8h, Persoons et al. (2017)). Both overall and pairwise estimates were computed according to Weir and Cockerham (1984).

The *Q*_ST_, an equivalent to *F*_ST_ for quantitative traits, was calculated for overall and for pairwise populations for each trait as defined by Spitze (1993):

$$Q_{\text{st}}=\frac{\sigma_{\text{Gb}}^{2}}{\sigma_{\text{Gb}}^{2}+2\sigma_{\text{Gw}}^{2}}$$

where *σ* ^2^_Gb_ is the quantitative genetic variance component between populations, and σ^2^_Gw_ is the quantitative genetic variance component within populations (corresponding to variance between strains), estimated from the full model described above.

To avoid misleading interpretation of *Q*_ST_–*F*_ST_ comparisons, confidence intervals of both indexes calculated on bootstrapped data have been taken into account (Leinonen et al., 2013). As a precaution, we considered *Q*_ST_ ≫ *F*_ST_ or *Q*_ST_ ≪ *F*_ST_. In these cases, it is likely that the trait has been under higher directional or stabilizing selection, respectively, compared with other traits in the same subset of populations (Firmat et al., 2017).

## 3 RESULTS

### 3.1 Phenotypic differentiation among populations

**Figure 1:**
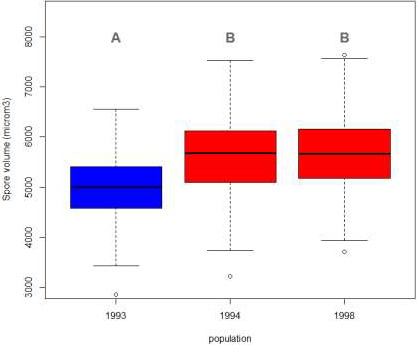
Box plot of spore volume (µm^3^) for each population. The blue box stands for avirulent “7” population sampled in 1993 and red boxes for virulent “7” populations sampled in 1994 and 1998. Letters correspond to Tukey's test results

Spore volume was the only trait to significantly differ among populations (Table 2), while none of the five aggressiveness traits varied consistently (Figure S2). Spore volume increased significantly following the resistance R7 breakdown event (*p*‐value < .0001, Figure 1). Strains from avirulent “7” population had a smaller volume (mean = 4,996 µm^3^) than strains from virulent “7” populations sampled after the resistance breakdown event in 1994 and 1998 (mean = 5,609 and 5,684 µm^3^ respectively). Considering sampling location (within temporal populations), we found that Belgian strains displayed intermediate values of spore volume (mean = 5,339 μm^3^): They were significantly bigger than 1993 strains but significantly smaller than French virulent “7” strains. Among strains originating from France, no significant difference was observed between 1994 and 1998 samples (Figure S3).

**Table 2:** Means and standard error of traits for each population

Trait	1993	1994	1998	ANOVA (*p*‐value)
Infection efficiency (%)	25 ± 1	30 ± 1	26 ± 1	1.000
Latent period (days)	7.23 ± 0.05	7.57 ± 0.06	7.26 ± 0.05	0.663
Lesion size (µm^2^)	520 ± 11	469 ± 10	479 ± 11	0.586
Mycelium quantity (AU)	3,455 ± 111	3,632 ± 122	3,120 ± 113	0.901
Sporulation rate (spore lesion^−1^ day^−1^)	238 ± 9	244 ± 8	250 ± 9	0.937
Spore volume (µm^3^)	4,996 ± 31	5,609 ± 36	5,684 ± 39	**9.00** × **10^–5^**

Note

*p*‐Value is reported for analysis of variance of models with and without the population term, in bold type where *p* < .05 after correction for multiple tests.

### 3.2 Heritability of measured traits

**Figure 2:**
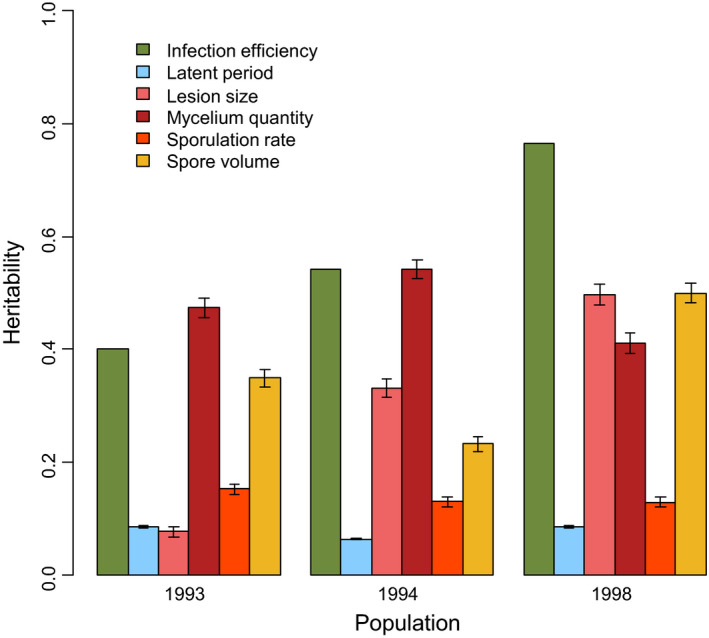
Heritability of aggressiveness and morphological traits for each population. The error bars represent 5% and 95% confidence intervals calculated from bootstrapped datasets (not available for infection efficiency)

In order to identify the traits potentially subjected to selection, we studied the heritability of each trait (Figure 2). This highlighted two main groups of traits: infection efficiency, spore volume, mycelium quantity, and lesion size had a relatively high heritability; and latent period and sporulation rate had much lower values.

According to the Kruskal–Wallis test followed by a post‐hoc Dunn test among populations, the heritability of infection efficiency and lesion size significantly increased between 1993 and 1998 from 0.40 to 0.77 and from 0.09 to 0.49, respectively. The heritability of *in planta* mycelium quantity was consistently high (>0.36). Spore volume also had a relatively high heritability (always > 0.24), which significantly increased between 1994 and 1998 to reach 0.47. On the contrary, the heritability of latent period and sporulation rate remained low (<0.14) across all populations.

### 3.3 *Q* _ST_‐ *F* _ST_ comparison for quantitative traits

**Figure 3:**
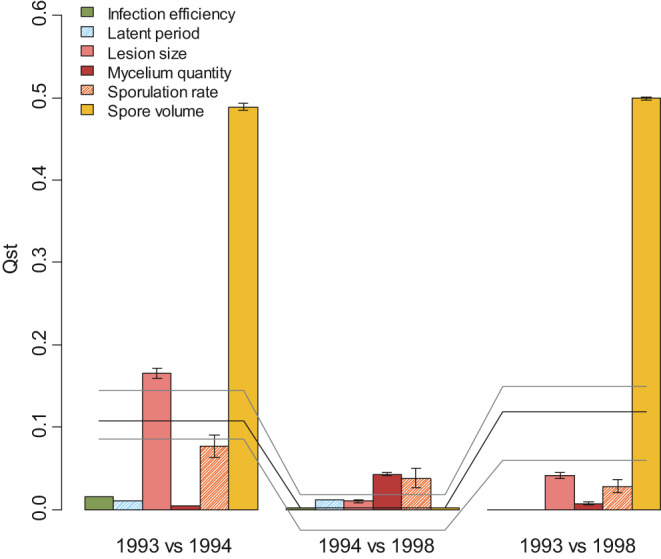
*Q*_ST_‐*F*_ST_ comparison. The bar plot represents *Q*_ST_ values (bars are grouped according to pairwise comparison and traits are displayed in the same order). Plain bars correspond to the most heritable traits and hatched bars to the less heritable ones. Confidence intervals (5% and 95%) were computed from bootstrap analysis (not available for infection efficiency). The black line corresponds to pairwise *F*_ST_ and gray lines represent 5 and 95% confidence intervals (jack‐knife over loci)

Overall, comparisons between avirulent “7” and virulent “7” populations (1993 vs. 1994 and 1993 vs. 1998) are similar: The *F*_ST_ values were around 0.10 and *Q*_ST_ values differed by traits (Figure 3). The *Q*_ST_ for spore volume reached 0.50, standing significantly above *F*_ST_. On the contrary, infection efficiency, latent period, and mycelium quantity showed very low *Q*_ST_ values (<0.015) and these *Q*_ST_ values were significantly lower than *F*_ST_. Lesion size and sporulation rate displayed *Q*_ST_ values nearly equal to the *F*_ST_ level (within confidence intervals). Comparing the two virulent “7” populations (1994 and 1998), no differentiation was observed, neither based on neutral markers (*F*_ST_ = 9.5 × 10^–4^) nor on quantitative traits.

### 3.4 Quantitative traits genetic correlations

**Figure 4:**
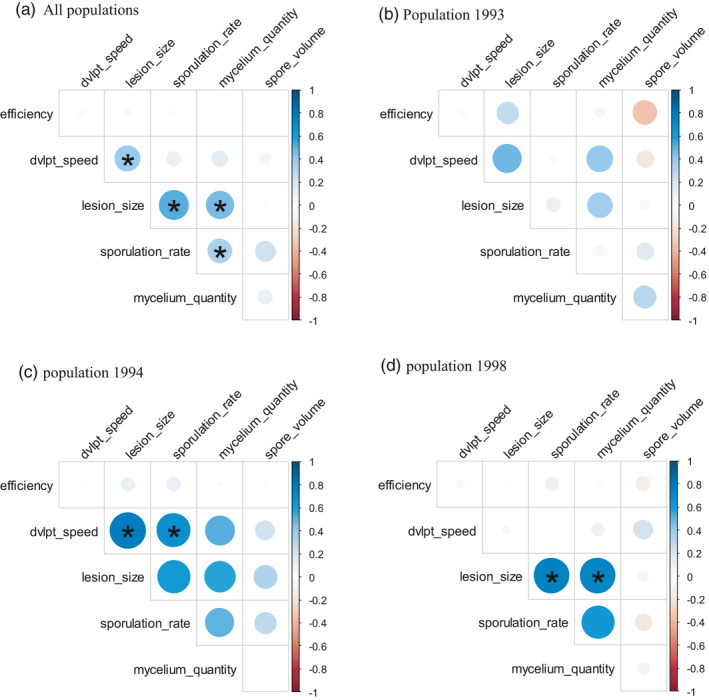
Pairwise Spearman correlation coefficients between fungal traits for all populations together (a) and each one separately (b: 1993; c: 1994; d: 1998). Colors correspond to the sign (blue: positive; red: negative) and dot size to the strength of the correlation. Stars indicate significant correlations according to Student's test adjusted with a false‐discovery rate correction for multiple tests. “dvlpt_speed” stands for development speed (inverse of latent period)

Genetic trade‐offs are reflected by negative genetic correlations between quantitative traits. Significant Spearman correlation coefficients calculated in this study were all positive, indicating that no trade‐offs shaped the studied traits (Figure 4).

However, positive genetic correlations have been highlighted. Over all populations, lesion size, development speed, sporulation rate, and mycelium quantity were positively correlated. These correlations were also found, in part, for populations 1994 and 1998. On the contrary, no significant correlation was found between traits in population 1993.

## 4 DISCUSSION

The aim of this study was to characterize the evolution of aggressiveness and morphological traits following the resistance R7 breakdown event. To test the hypothesis of evolution driven by this event, we measured six quantitative traits on three *M. larici‐populina* populations in a common garden experiment using a single‐spore inoculation protocol.

Among the six traits measured (infection efficiency, latent period, lesion size, mycelium quantity, sporulation rate, and spore volume), spore volume was the only one to vary significantly among the three populations. Other traits, usually associated with aggressiveness, were constant. In the following discussion, we discuss traits’ evolution (or the lack thereof) following a major qualitative resistance breakdown event, considering their heritability, the selection applied on them and their genetic covariances.

### 4.1 Change in spore volume at the time of the resistance R7 breakdown event

The observed increase in spore volume in 1994 is likely to be a consequence of the resistance R7 breakdown event. Indeed, the change in spore volume is associated to a high heritability and a potential directional selection at the time of the breakdown event, providing evidence for evolution following this resistance breakdown. The heritability values of spore volume we found are not surprising as Stefansson et al. (2014) estimated a comparable heritability of spore size (0.55) in the barley scald pathogen, *Rhynchosporium commune*. Additionally, *Q*_ST_–*F*_ST_ comparison between populations from 1993 and 1994 indicated the presence of directional selection acting on spore volume. This could imply a rapid response to selection for this trait. A similar result in the short‐term evolution of spore size and shape has been observed by Delmas et al. (2016) as an adaptation to quantitative resistance in grapevine downy mildew. Bever and Morton (1999) and Bentivenga et al. (1997) also observed a rapid evolution of these morphological traits in a single generation of selection on arbuscular mycorrhizal fungi.

The spread to new susceptible hosts after a qualitative resistance breakdown necessarily implies dispersal of the pathogen. It has been shown that poplar rust spores have long‐distance dispersal abilities (Barrès et al., 2008). The observed evolution of spore volume could thus reflect an adaptation to dispersal resulting from the rapid invasion of virulent “7” strains across France (Xhaard et al., 2011). In a study conducted in the Durance River valley (Pernaci, 2015), we indeed showed that migrant spores tended to have a larger volume than resident ones. The first virulent “7” strains were found in Belgium in September 1994 and subsequently sampled in northern France (Pinon & Frey, 2005). In accordance with this scenario, we observed intermediate values of spore volume in Belgian strains.

### 4.2 No variation of aggressiveness traits following resistance R7 breakdown

Among all quantitative traits we measured, the volume of spores was the only trait to evolve following the resistance R7 breakdown event. All strains were assayed from the same single‐spore inoculations in a balanced design. We thus argue that the lack of variation of aggressiveness traits is a robust experimental result with biological significance.

The lack of evolution of aggressiveness traits in a four‐year period following the resistance breakdown is consistent with the theoretical results of Gandon and Michalakis (2000). This modeling study showed that qualitative host resistance triggers a relatively slow decrease of aggressiveness. Conversely, quantitative resistance—in particular if this resistance targets within‐host multiplication—is predicted to increase aggressiveness because it directly influences the trade‐off between parasite transmission and growth. Even if there is a strong selection pressure exerted on pathogen populations during a qualitative resistance breakdown, it does not directly affect aggressiveness traits.

In case of resistance breakdown, a virulence cost can be expected because the acquisition of a virulence factor may be penalized by a decrease in fitness resulting from the loss of the avirulence gene function (Papaïx et al., 2018; Pariaud et al., 2009). Here, we did not observe such a fitness cost: Aggressiveness was not changed after a virulence gain. Controlled experiments reduce environmental variation and can thus mask fitness differences that result from stress occurring under natural conditions (Laine & Barrès, 2013). In particular, the single‐spore inoculation protocol prevented interactions between spores where co‐infection could affect fungal fitness. Also, under natural conditions, *M. larici‐populina* epidemics involve a wide set of traits which this study did not consider, such as overwinter survival or success of sexual reproduction on larch. Unfortunately, we are not yet able to reproduce the whole life cycle of this pathogen in a laboratory setting to measure these quantitative traits precisely. Even at the stage of uredinia, the set of traits considered in this study was not exhaustive. Thus, fitness costs could also be undetectable if compensatory mechanisms benefit virulent strains and restore fitness (Laine & Barrès, 2013).

It is noteworthy that our experiment has been undertaken with only one susceptible poplar cultivar (‘Robusta’) since we aimed to compare virulent and avirulent strains on the same cultivar. To further investigate the evolution of aggressiveness traits following a resistance breakdown event, it could be worth measuring the same set of traits on other poplar cultivars to test for host adaptation.

### 4.3 Contrasted heritability of aggressiveness traits

The lack of evolution of aggressiveness traits contrasts at first glance with the high heritability values observed for most traits, especially lesion size and mycelium quantity. A high heritability is a prerequisite for an evolutionary response to selection on traits, as we observe for spore volume. We even observe an increase through time in heritability of lesion size. However, the *Q*_ST_‐*F*_ST_ comparisons indicate that these trait values, especially mycelium quantity, are conserved through time. The conservation of these traits at the time of the resistance R7 breakdown event may be a consequence of a stabilizing selection or of a canalization phenomenon, which could indicate evolutionary constraints on infection.

It should be noted that we have estimated heritability from measurements in a laboratory experiment, probably leading to an overestimation of heritability values in comparison with field estimates (Charmantier & Garant, 2005). Nevertheless, relatively high heritability of aggressiveness traits has been observed in several crop pathogens such as the barley pathogen *Rhynchosporium commune* (Stefansson et al., 2014), the wide host range pathogen *Rhizoctonia solani* (Willi et al., 2011), the corn pathogens *Helminthosporium maydis* (Hill & Nelson, 1982), and *Cochliobolus carbonum* (Hamid et al., 1982), or the wheat pathogen *Zymoseptoria tritici* (Willi et al., 2010).

On the contrary, we observed very low heritability values for two traits: sporulation rate and latent period, whatever the population considered. These low heritability values may be consistent with the study of Price and Schluter (1991) who concluded that morphological traits, for example, spore volume, tend to have a higher heritability than life‐history traits. Among aggressiveness traits, sporulation rate and latent period are most likely to be thought of as life‐history traits. Latent period, which is the onset of sporulation, amounts to the age at reproduction in plants and animals. Sporulation rate determines the number of propagules that a genotype can produce which directly influence transmission and pathogen fitness.

### 4.4 Changes in genetic correlations following the resistance R7 breakdown

While trade‐offs among life‐history and/or aggressiveness traits have been demonstrated in other fungal plant pathogens (Lannou, 2012; Pariaud et al., 2009), we found no evidence of trade‐offs in our dataset. Conversely, we observed positive genetic correlations.

Considering all populations together, significant genetic correlations between mycelium quantity, lesion size, and sporulation rate were observed. These positive correlations may reflect differences of fitness between strains within populations (Nespolo et al., 2014), in that some strains have increased growth: A greater mycelium quantity may produce more spores, leading to wider lesion size on the leaf surface. It must be emphasized that these significant positive correlations may prevent us from detecting trade‐offs (Fry, 1993).

Among all populations, the strongest correlation was found between lesion size and sporulation rate. The correlation between lesion size and spore production has already been observed at a phenotypic level in Dowkiw et al. (2003) using a strain of *M. larici‐populina* from 1993 on 336 poplar genotypes. Maupetit et al. (2018) also highlighted a positive correlation between lesion size and sporulation rate with a strain from 1998. The correlation was described as a response to an environmental gradient (leaf maturity gradient), hence resulting from plasticity. The study described here, including three populations, allows us to generalize the correlation and to add a genetic determinism linking lesion size and sporulation rate.

Genetic correlations changed through time and, interestingly, involved different traits in 1994 and 1998. Only correlations involving lesion size and sporulation rate were significant after the resistance R7 breakdown. Simultaneously, we observed an increase in spore volume. Altogether this points to the fact that dispersal was a key process at that time, which is consistent with a period of invasion of an ecological niche during host range expansion.

## 5 CONCLUDING REMARKS

In summary, the temporal sampling studied in this work allowed us to document a probable evolution of spore volume under directional selection concomitant to the resistance R7 breakdown event. In contrast, aggressiveness traits did not differ significantly. In spite of the strong selection pressure exerted on pathogen populations during resistance breakdown, this event did not detectably affect the evolution of aggressiveness traits.

Nowadays, breeders focus more and more on quantitative resistance because they are thought to be more durable. However, several studies have highlighted that quantitative resistance can be eroded leading to the increase of aggressiveness (Andrivon et al., 2007; Caffier et al., 2014; Cowger & Mundt, 2002; Delmas et al., 2016). Should our results be generalized to most cases of qualitative resistance breakdown, we might conclude that the eventual breakdown of qualitative resistance deployment would be less detrimental to sustainable crop production than breakdown of quantitative resistance traits. While one type of qualitative resistance was overcome, it did not lead to a population‐level increase in parasite aggressiveness. The most promising strategies in sustainable production thus lie in rational deployment of qualitative resistances, such as pyramiding resistances or spatio–temporal arrangement in the mosaic of agricultural landscape (Djidjou‐Demasse et al., 2017).

## 6 DATA ARCHIVING STATEMENT

Data and R scripts are available at the Dryad Digital Repository: https://doi.org/10.5061/dryad.j6q573nbw

## Conflict of interest

None declared.

## Supporting information

Supplementary Material
