# Supplementary material for: Evolution of morphological but not aggressiveness‐related traits following a major resistance breakdown in the poplar rust fungus, Melampsora larici‐populina
Source: Evol Appl. 2020 Oct 16;14(2):513–23. doi: 10.1111/eva.13136 (PMC7896724; doi:10.1111/eva.13136)

Supporting information

Figure S1: Incomplete block design for qPCR analysis

qPCR run number	DNA extraction plate number																		
	**1**	**2**	**3**	**4**	**5**	**6**	**7**	**8**	**9**	**10**	**11**	**12**	**13**	**14**	**15**	**16**	**17**	**18**	**19**
**1**	1	2	3	4															
**2**					5	6	7	8											
**3**									9	10	11	12							
**4**													13	14	15	16			
**5**	1																17	18	19
**6**		2				6				10				14					
**7**			3				7				11				15				
**8**				4				8				12				16			
**9**					5				9				13				17		
**10**		2					7					12					17		
**11**			3					8					13					18	
**12**	1				5					10					15				
**13**				4					9					14					19
**14**						6					11					16			19
**15**																		18	
Total No of repetition per plate	3	3	3	3	3	3	3	3	3	3	3	3	3	3	3	3	3	3	3

Figure S2: Barplot of infection efficiency (%) with standard errors and boxplots of latent period (days), lesion size (µm^2), mycelium quantity and sporulation rate (spore/day) for each population. Blue box stands for avirulent population sampled in 1993 and red boxes for virulent populations sampled in 1994 and 1998.

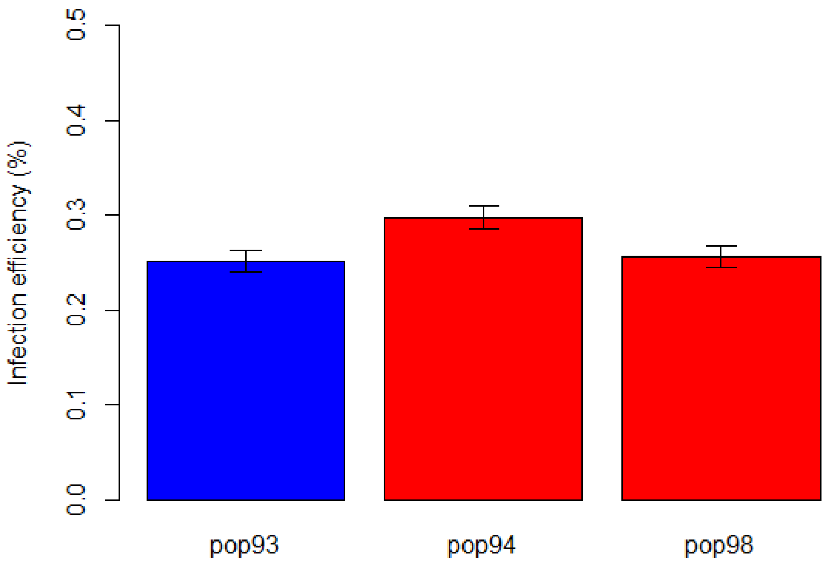

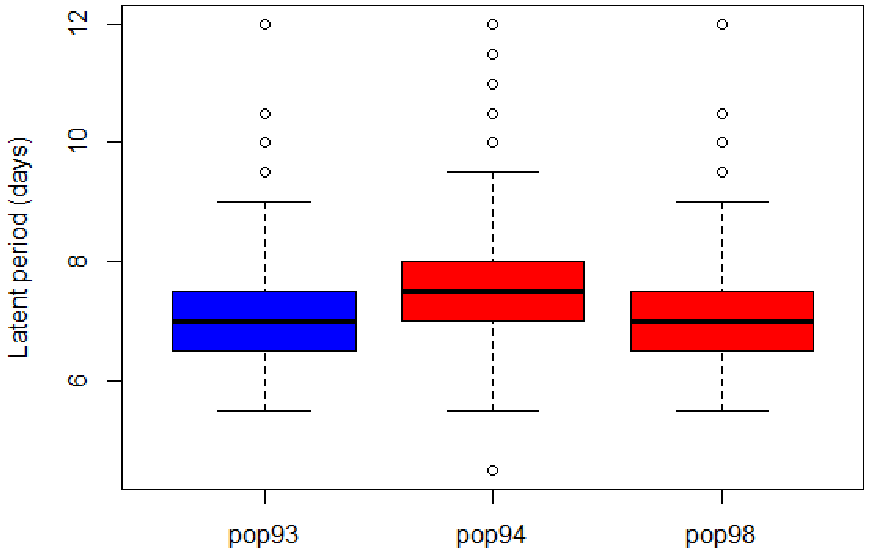

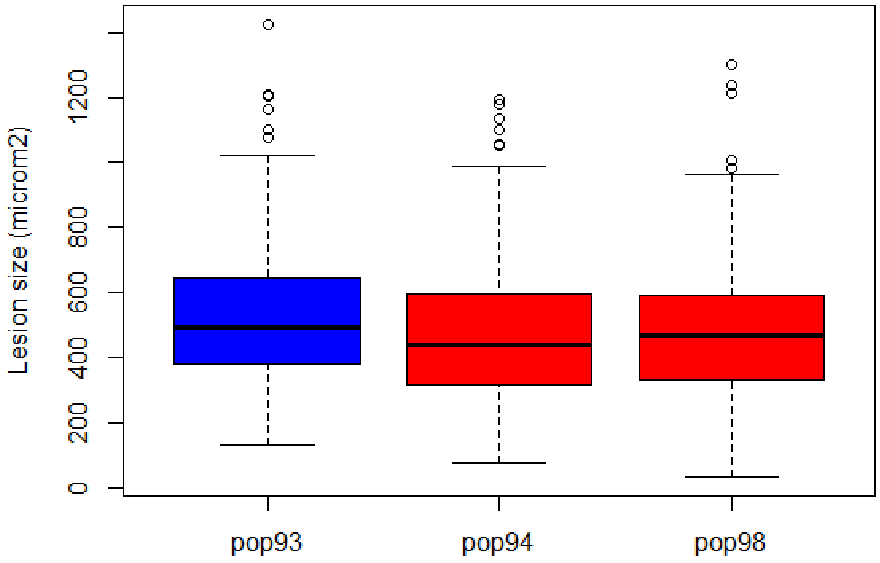
Lesion size (microm2)
0
200
400
600
800
1200
pop93
pop94
pop98

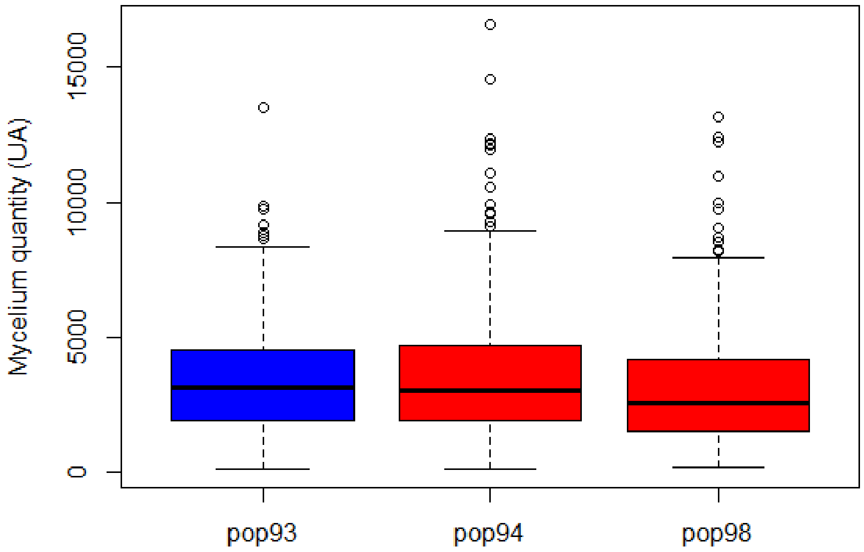
Mycelium quantity (UA)
0
5000
10000
15000
pop93
pop94
pop98

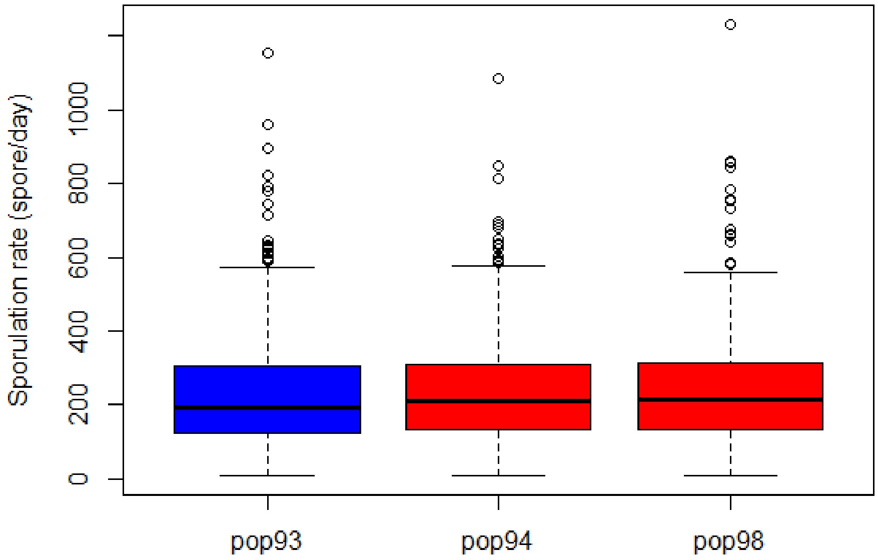
Sporulation rate (spore/day)
0
200
400
600
800
1000
pop93
pop94
pop98

Table S1: Strains and their respective population, sub-population and sampling location (in brackets: French departement zip code).

Strain	Population	Sub population	Location
93CV1	pop93	pop93	Amance (54)
93DL2	pop93	pop93	Amance (54)
93EA2	pop93	pop93	Amance (54)
93EC4	pop93	pop93	Amance (54)
93EF8	pop93	pop93	Amance (54)
93GR7	pop93	pop93	Amance (54)
93GS3	pop93	pop93	Amance (54)
93GT1	pop93	pop93	Amance (54)
93GV8	pop93	pop93	Amance (54)
93GW3	pop93	pop93	Amance (54)
93HW5	pop93	pop93	Amance (54)
93IB7	pop93	pop93	Amance (54)
93IC8	pop93	pop93	Amance (54)
93NU2	pop93	pop93	Amance (54)
93NU4	pop93	pop93	Amance (54)
93NX6	pop93	pop93	Amance (54)
93OC5	pop93	pop93	Amance (54)
93Q1	pop93	pop93	Amance (54)
94ZZ3	pop94	pop94_Be	Grammont (Belgium)
94ZZ33	pop94	pop94_Be	Grammont (Belgium)
94ZZ35	pop94	pop94_Be	Grammont (Belgium)
94ZZ36	pop94	pop94_Be	Grammont (Belgium)
94ZZ37	pop94	pop94_Be	Grammont (Belgium)
94ZZ7	pop94	pop94_Be	Grammont (Belgium)
94ZZ1	pop94	pop94_Fr	Chereng (59)
94ZZ10	pop94	pop94_Fr	Onnang (59)
94ZZ11	pop94	pop94_Fr	Lecelles (59)
94ZZ12	pop94	pop94_Fr	Bucy-les-cerny (02)
94ZZ13	pop94	pop94_Fr	Hesdin (62)
94ZZ14	pop94	pop94_Fr	Guesnain (59)
94ZZ15	pop94	pop94_Fr	Saulchoy (62)
94ZZ16	pop94	pop94_Fr	Saulchoy (62)
94ZZ18	pop94	pop94_Fr	Etain (55)
94ZZ19	pop94	pop94_Fr	Francilly - Selency (02)
94ZZ20	pop94	pop94_Fr	Nogent (45)
94ZZ21	pop94	pop94_Fr	Chereng (59)
98AB02	pop98	pop98_FrAm	Amance(54)
98AC08	pop98	pop98_FrAm	Amance(54)
98AD04	pop98	pop98_FrAm	Amance(54)
98AP2	pop98	pop98_FrAm	Amance(54)
98AP3	pop98	pop98_FrAm	Amance(54)
98GB3	pop98	pop98_FrAm	Amance(54)

98GD5	pop98	pop98_FrAm	Amance(54)
98GF7	pop98	pop98_FrAm	Amance(54)
98AG18	pop98	pop98_FrMoy	Moy-de-l'Aisne (02)
98AG3	pop98	pop98_FrMoy	Moy-de-l'Aisne (02)
98AG31	pop98	pop98_FrMoy	Moy-de-l'Aisne (02)
98AG36	pop98	pop98_FrMoy	Moy-de-l'Aisne (02)
98AG42	pop98	pop98_FrMoy	Moy-de-l'Aisne (02)
98AG49	pop98	pop98_FrMoy	Moy-de-l'Aisne (02)
98AG54	pop98	pop98_FrMoy	Moy-de-l'Aisne (02)
98AG69	pop98	pop98_FrMoy	Moy-de-l'Aisne (02)
98AI1	pop98	pop98_FrMoy	Moy-de-l'Aisne (02)
98AI58	pop98	pop98_FrMoy	Moy-de-l'Aisne (02)

Figure S3: Boxplot of spore volume (μm^3) for each sub-population. The blue box stands for avirulent 7 population sampled in 1993 and red boxes for virulent 7 populations sampled in 1994 and 1998. Letters correspond to Tukey test results.

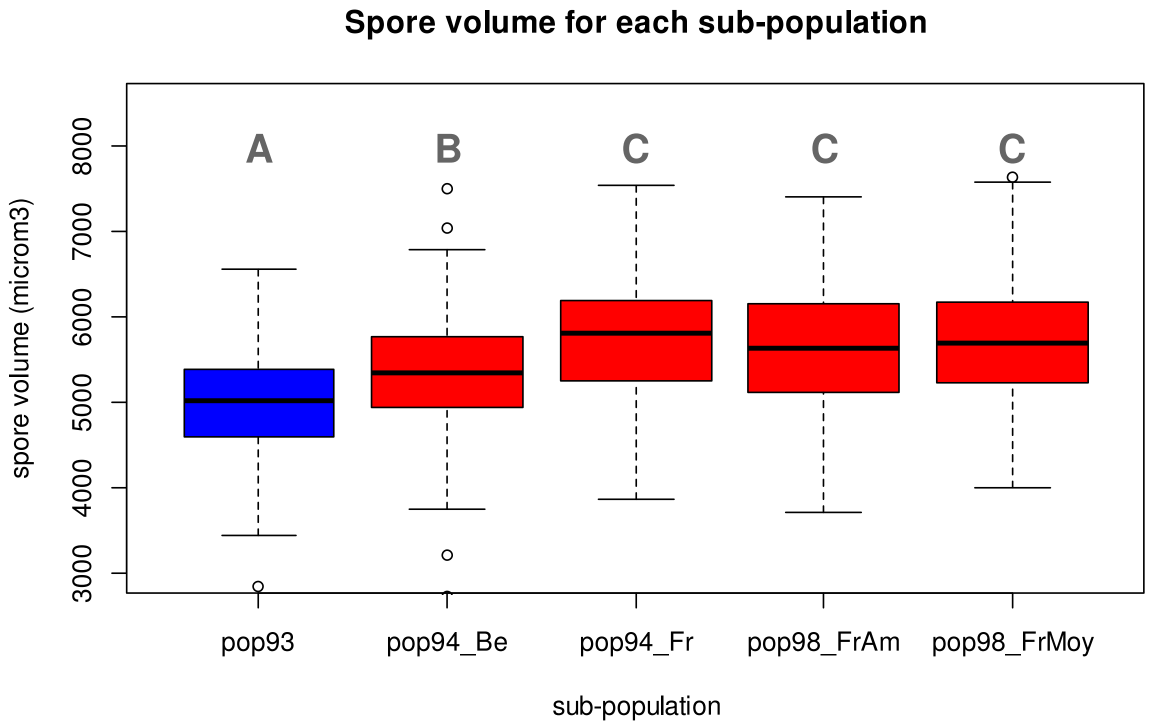

Supplement: Supplementary file 1 — Supplementary Material [file EVA-14-513-s001.pdf]
